# Collagen type I mimicking peptide additives to functionalize synthetic supramolecular hydrogels

**DOI:** 10.1016/j.mtbio.2024.101021

**Published:** 2024-03-15

**Authors:** Annika F. Vrehen, Johnick F. van Sprang, Maaike J.G. Schotman, Patricia Y.W. Dankers

**Affiliations:** Institute for Complex Molecular Systems, Department of Biomedical Engineering, Laboratory of Chemical Biology, Eindhoven University of Technology, Groene Loper 7, Eindhoven, 5612, AZ, the Netherlands

**Keywords:** Supramolecular chemistry, Hydrogels, Collagen mimics, Peptide additives

## Abstract

Small bioactive peptide sequences derived from extracellular matrix proteins possess the ability to interact with cell receptors. As such, these peptide additives are excellent mimics to develop materials for 3D cell culture. Two types of supramolecular modified collagen type I mimicking peptide additives are presented; UPy-GFOGER (39 amino acids), with a novel superstructure, and the more simplistic UPy-DGEA (7 amino acids). Here, we studied the impact of the conformational differences between both peptide additives, on their biological performance. Various analyzing techniques demonstrated the ability of the supramolecular UPy-GFOGER to self-assemble into short nanofibers with brush-like outer features, suggesting trimerization into a triple helix. UPy-DGEA is a short additive without a complex structure. Since, collagen type I is a major component of the human corneal stroma, primary keratocytes (PKs) are encapsulated within the functionalized hydrogels to provide insights in the induced bioactivity of both additives. Incorporation of UPy-GFOGER supported an elongated morphology and (re-)differentiation of the encapsulated PKs, while tiny round-shaped cells were observed within the hydrogels functionalized with UPy-DGEA. This difference in biological success between UPy-GFOGER and UPy-DGEA indicates the difficulty of using short peptide additives without a complex structure to mimic the complex structure of natural collagen.

## Graphical abstract

**Figure:**
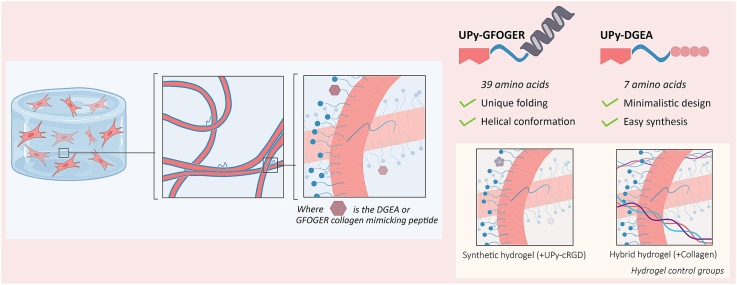

## 1 Introduction

Via various receptors a cell is continuously interacting with its own specific native microenvironment, the extracellular matrix (ECM) [1]. Collagens are the most abundantly present proteins within the ECM, forming supramolecular assemblies that participate in cell-matrix interactions. Based on their supramolecular assemblies collagens can be subdivided into various subfamilies such as fibrils, beaded filaments, anchoring fibrils, and networks. The human cornea is located at the outermost part of the eye, covering the tear film. The thickest layer within the cornea is the stroma, which due to its size substantially affects the corneal functions. An important characteristic of the stroma is its very specific and highly organized structure of packed collagen type I fibrils. These fibrils are surrounded by specialized proteoglycans, resulting in a highly hydrated tissue [2,3]. A collagen type I molecule consists of a typical (Gly-X-Y)_n_ repeating sequence with the variable amino acids often being a proline or hydroxyproline. This specific repeating sequence induces a triple-helical conformation [[4], [5], [6]]. Via *cis*- or trans peptide bonds between the proline residues polyprolines are known to from either a right-handed polyproline I helix or a left-handed polyproline II helix [[7], [8], [9]]. Three α chains of collagen type I in a left-handed polyproline II-helix conformation coil around each other to assemble into a right-handed triple helix [4,8,[10], [11], [12]]. The self-assembly of collagen into a triple helix conformation is stabilized through hydrogen bonding between N–H groups of the glycines and C=O groups of the prolines or hydroxyprolines [8,10,13]. Cell-matrix interactions are initiated via short bioactive sequences of amino acids, derived from various ECM proteins in the microenvironment surrounding the cells that enable binding to cellular receptors [[14], [15], [16], [17], [18]]. Via solid-phase peptide synthesis (SPPS) it is possible to synthesize these short amino acid sequences, whereafter they can be used to functionalize biomaterials. Previously, various short collagen mimicking peptides have been generated, *i.e.* RGDS (present in laminin, collagen I and IV) [19,20], DGEA (present in collagen I and IV) [21,22], GTPGPQGIAGQRGVV (linear peptide derived from the α1 helix of collagen I) [23], RADA16-GG-FPGERGVEGPGP (derived from collagen I) [24], GGYGGGPC(GPP)5-GFOGER-(GPP)_5_GPC (derived from collagen I) [25], and (PKG)_4_(POG)_4_(QOG)_4_ (derived from collagen I) [26]. In addition, some studies demonstrated the need of a triple-helical conformation of GFOGER-like additives to allow improved bioactivity upon usage in biomaterials. The GFOGER sequence is often flanked by Gly-Pro-Pro (GPP) repeating units to induce trimerization into a triple-helical conformation [10,[27], [28], [29], [30]].

The incorporation of peptide additives into a supramolecular system allows the material to mimic the dynamic and reconfigurable nature of the ECM. For instance, the use of peptide amphiphiles (PA), which possess the ability to interact with cells through a high density of surface signals. Cell encapsulation within the three-dimensional supramolecular network of PA nanofibers is achieved via the presentation of a bioactive epitope on the nanofiber surface. Various short peptide sequences have been used as bioactive epitope, such as the laminin derived IKVAV sequence or VEGF-mimetic peptide sequences [[31], [32], [33]]. Besides PAs, supramolecular hydrogels are also formed from molecules with bifunctional or monofunctional fourfold hydrogen bonding designs. The use of bifunctional and monofunctional molecules containing ureido-pyrimidinone (UPy) groups as supramolecular building blocks was studied by Diba et al., demonstrating dynamic hydrogels functionalized with cyclic integrin-binding arginine-glycine-aspartate (cRGD) ligands to enhance cell spreading [34]. To effectively present the cRGD ligand to the cells an UPy-moiety was modified with cRGD to allow incorporation into the monofunctional fiber. While non-modified cRGD additives showed to be ineffective to promote cell attachment, since the molecules were prone to burst release [34]. Previous studies focusing on the peptide presentation within an UPy-network demonstrated the need of peptides conjugated to UPy-moieties as well. Together, these studies resulted in an amphiphilic design, in which a short oligo(ethylene glycol)(OEG) spacer was introduced [[35], [36], [37], [38], [39], [40], [41]]. UPy-dimers self-assemble into 1D stacks, using π-π interactions stabilized by quadruple hydrogen bonding via a flanking urea group, which allows these stacks to turn into a bundle [34,36,42,43]. Conjugation of the synthesized peptides to a supramolecular UPy-moiety allows, due to the modularity of supramolecular materials, the formation of a stable bulk material functionalized with these peptide additives, acting as biochemical cues to induce cell adhesion, cell spreading, or even more complex cell-material interactions [35,[37], [38], [39], [40], [41],[44], [45], [46], [47], [48], [49]].

**Fig. 1:**
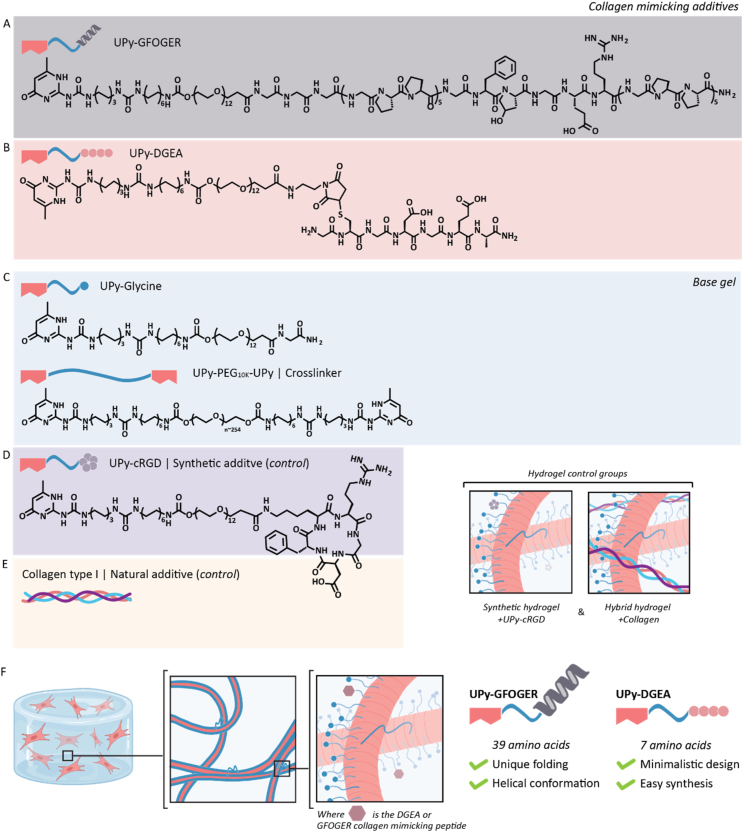
Chemical structures of the molecules and schematic representation of the studied conditions. **A)** The chemical structure of UPy-GFOGER. **B)** The chemical structure of UPy-DGEA. **C)** The chemical properties of the hydro gelators and a schematic representation of the fiber assembly and hydrogel formation. **D)** The chemical structure of UPy-cRGD. **E)** Schematic representation of collagen type I, used within the hybrid hydrogel. **F)** Schematic illustration of the supra molecular network. Blue linkages between fibers indicate interfiber cross-links formed by UPy-PEG_10K_-UPy. The collagen mimicking additives UPy-GFOGER and UPy-DGEA are used to functionalize a synthetic supramolecular hydrogel. (For interpretation of the references to colour in this figure legend, the reader is referred to the Web version of this article.)

Here, a collagen type I mimicking polypeptide GGG-GPP_5_-GFOGER-GPP_5_ (GFOGER) (Fig. 1A) and a short GCGDGEA (DGEA) peptide (Fig. 1B) are synthesized, conjugated to an UPy-moiety and studied for their bioactivity when incorporated within an UPy-based hydrogel. The fundament of the supramolecular UPy-based hydrogel is based on a 528 Da oligo(ethylene glycol) (OEG) chain end-capped with a UPy moiety at one end and a glycine-amide group at the other end (UPy-Glycine) and a 10 kDa poly(ethylene glycol) (PEG) chain, end-capped with two functional UPy-moieties (UPy-PEG_10K_-UPy) (Fig. 1C). An alkyl spacer is incorporated to shield the hydrogen bonds in water, to create a hydrophobic pocket upon assembly of UPy-dimers into 1D stacks. Hydrogen bonding of flanking urea groups implements further stabilization of the formed stacks, and further assembly occurs by bundling of the stacks into fibers. Due to the molecular design of the UPy-Glycine it is expected that the glycols’ non-fouling properties are shielded from exposure, while the glycine-amide groups are assumed to be presented to the cells. Upon addition of the UPy-PEG_10K_-UPy interfiber cross-links are formed, resulting in the formation of a hydrogel [34,50,51]. Two control hydrogels were incorporated in this study as a reference, namely a synthetic UPy hydrogel functionalized with UPy-cRGD (Fig. 1D) and a hybrid hydrogel based on a mixture of UPy molecules and collagen type I (Fig. 1E) [51]. While both peptide additives are designed to mimic collagen type I, we assume only the UPy-GFOGER additive will fold into a supramolecular aggregate comprising a triple-helical conformation. This assumption is based on the presence of GPP repetition units within the UPy-GFOGER, and the absence of these units within the UPy-DGEA. Within this study we will investigate the influence of this conformational difference between the additives on their bioactivity, when both are individually used to functionalize a synthetic hydrogel (Fig. 1F). Since collagen type I is major component of the human corneal stroma, primary corneal keratocytes are encapsulated and cultured within UPy-GFOGER and UPy-DGEA functionalized hydrogels to provide insights about the induced bioactivity of both additives [52,53].

## 2 Results and discussion

### 2.1 Synthesis of the bioactive GFOGER additive and conjugation to an UPy-moiety

N- or C-terminus modifications of the collagen type I-mimicking peptide GGG-GPP_5_-GFOGER-GPP_5_ (GFOGER) with additional supramolecular moieties, e.g. aromatic, metal-coordinating or electrostatic groups, allowed for the generation of higher-ordered structures [5,11,[54], [55], [56], [57]]. Here, an UPy-moiety was conjugated to the GFOGER polypeptide N-terminus (UPy-GFOGER) whereafter the self-assembly behavior with respect to triple helix formation and fiber formation was explored.

Standard Fmoc SPPS on a rink amide resin was performed for the synthesis of the GFOGER polypeptide. After each amino acid coupling an acetylation step was performed to prevent the growing of unreacted peptide chains that lack one or more amino acids. The Fmoc protecting the N-terminus of the polypeptide was removed by using the base piperidine (SI, Scheme S1). Subsequently, the polypeptide was deprotected and removed from the resin using a cleaving mixture of trifluoroacetic acid (TFA) and ultrapure water, mixed with triisopropylsilane (TIS). Due to the growth of peptide chains that did not react during an amino acid coupling step on the resin, synthesizing polypeptides in high purity using SPPS was considered to be challenging. Some impurities are detected after cleaving the peptide from the resin, whereafter the GFOGER polypeptide was purified using preparative RP-LCMS. To this end, the polypeptide was gained with a yield of 19% in high purity (>95%). An UPy-synthon with an UPy-C6-U-C12-Uret-mOEG12-C2-COOH molecular design was used to modify the N-terminus of the GFOGER polypeptide, creating a supramolecular collagen mimicking bioactive additive. The use of HATU and DIPEA activated the carboxylic acid group, which reacted with the primary amine located at the N-terminus of the GFOGER polypeptide. Residual UPy-synthon was removed by running a preparative RP-LCMS, which decreased the final yields due to the affinity of the OEG-segment for the silica column. However, UPy-GFOGER is successfully synthesized and gained with a yield of 13% in high purity (>95%).

### 2.2 Synthesis of the bioactive GCGDGEA additive and conjugation to an UPy-moiety

The GCGDGEA peptide was synthesized using standard Fmoc solid phase peptide synthesis (SPPS) on a rink amide resin, with a propargyl glycine at the N-terminus and an introduced cysteine on the C-terminus to enable coupling to the UPy-moiety. The Fmoc-protected GCGD(tBU)GE(tBU)A peptide was deprotected using piperidine as a base, resulting in a primary amine. Thereafter, the peptide was deprotected and removed from the resin using TFA and ultrapure water mixed with TIS to simultaneously remove the side-protective groups on the amino acids (SI, Scheme S2). Upon using TFA, highly reactive cationic species were generated from the protecting groups that could react with the nucleophilic group of the cystine, the scavenger ethanedithiol (EDT) was added to quench these ions. Residual unreacted peptides were removed through purification with preparative reverse-phase liquid chromatography-mass spectrometry (RP-LCMS). The GCGDGEA peptide was attached to the UPy-moiety via maleimide-thiol chemistry, where Tris(2-carboxyethyl)phosphine (TCEP) was used to cleave the sulfide bonds and therewith activate the thiol in the cysteine to react with the maleimide. The UPy-GCGDGEA was gained with a yield of 54% in high purity (>96%).

### 2.3 The ability of UPy-GFOGER to self-assemble: the effects of diluting and mixing on triple helix formation

**Fig. 2:**
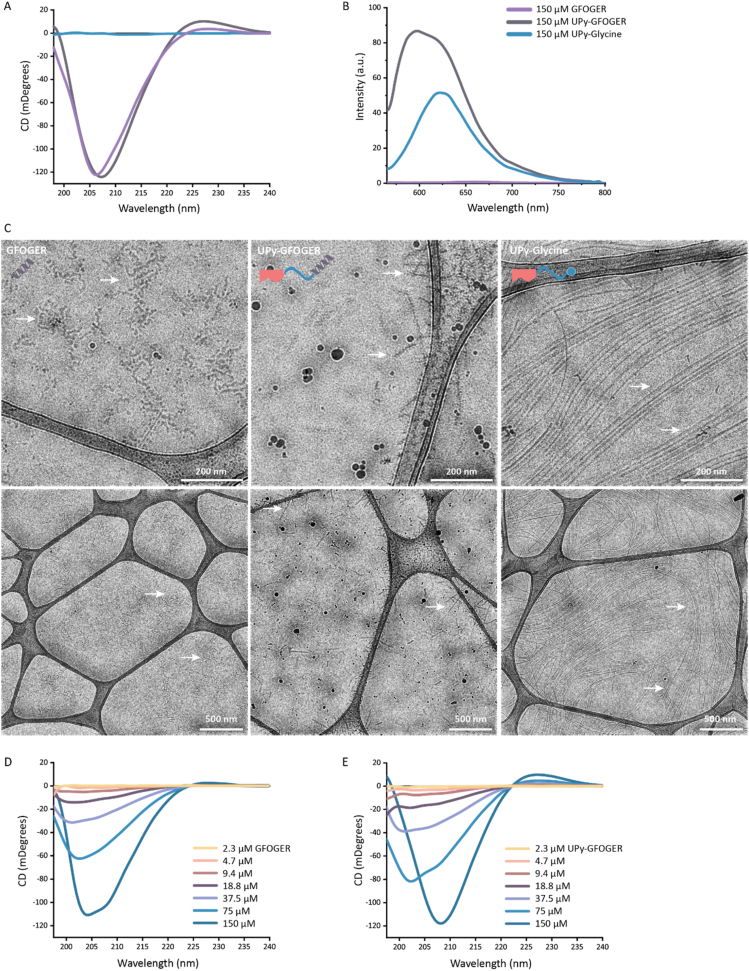
Morphological analysis of supra molecular assemblies containing triple helix forming regions. **A)** CD spectra of GFOGER, UPy-GFOGER, and UPy-Glycine at a concentration of 150 μM at 37 °C. **B)** Fluorescent emission spectra of Nile red dye (7.5 μM) in GFOGER, UPy-GFOGER, and UPy-Glycine solutions (150 μM) at 37 °C. **C)** Cryogenic transmission electron micrographs of supramolecular assemblies of GFOGER, UPy-GFOGER, and UPy-Glycine. **D)** CD spectra of dilution series of GFOGER at 37 °C. **E)** CD spectra of dilution series of UPy-GFOGER at 37 °C. (For interpretation of the references to colour in this figure legend, the reader is referred to the Web version of this article.)

The combination of the dihedral bond angle in the repeating glycine-proline-proline pattern of the GFOGER polypeptide, together with the intermolecular hydrogen bonding between the amide groups induces trimerization into a triple helix conformation in an aqueous environment [5]. The triple helix conformation associates with a specific optical activity, allowing circular dichroism (CD) spectroscopy to investigate the presence of a helical formation [58]. GFOGER, the UPy-GFOGER conjugate, and UPy-Glycine (functioning as a control) were deprotonated and dissolved in alkaline PBS and afterwards neutralized to a pH of ∼7.4 with HCl solution. Overnight molecular assembly was allowed at 4 °C to ensure triple helix formation, and optical activity was measured at 37 °C. UPy-Glycine did not show any CD (Fig. 2A). Both GFOGER as well as UPy-GFOGER exhibited spectral features characteristic of a triple helix, showing maxima around 225 nm, a crossover around 220 nm, and a broad negative peak near 205 nm [58]. These results indicate that the CD behavior of UPy-GFOGER is caused by the polypeptide segment.

The observed fiber-like morphology within the UPy-GFOGER assemblies could be caused by stacking of UPy-dimers. To investigate this scenario, a Nile red dye, which only fluoresces in a hydrophobic environment, is used. Nile red has an excitation wavelength of 485 nm, while the emission wavelength is mainly dependent on the polarity of the environment. If the lipophilic dye incorporates as a guest molecule into the hydrophobic core of a self-assembled UPy-fiber, a fluorescent signal can be observed [59,60]. UPy-Glycine showed a distinct fluorescent emission spectrum with a peak at a wavelength of 620 nm (Fig. 2B), indicating a hydrophobic pocket in the UPy-fiber for the Nile red dye to incorporate into. The fluorescent emission spectrum of UPy-GFOGER showed a blue shift as well as a broader shoulder between 600 and 625 nm, indicating an increase in hydrophobicity of the environment for the Nile red dye and stacking of the UPy-GFOGER molecules [59,60].

Visualization of the morphologies of the self-assembled structures of GFOGER, UPy-GFOGER, and UPy-Glycine are demonstrated by cryogenic transmission electron microscopy (cryo-TEM) images (Fig. 2C). In aqueous environment, the GFOGER polypeptide formed small aggregates with coiled features. A quite different visualization was observed for the UPy-GFOGER molecules that assembled into small nanofibers with a distinct brush-like morphology, showing a preference for localization around the carbon grid. Similar to previous work, UPy-Glycine molecules aggregated into very long nanofibers with a smooth appearance compared to the brush-like UPy-GFOGER aggregates [34]. These results suggest that the brush-like structures observed around the UPy-GFOGER fibers originate from triple helices formed by the GFOGER polypeptide segments.

Two segments within UPy-GFOGER allow for self-assembly of the molecule: the dimerizing UPy-moiety (1), and the trimerizing GFOGER polypeptide region (2). To study if one of these two segments dominates the assembly behavior of UPy-GFOGER, dilution series were prepared of GFOGER and UPy-GFOGER ranging from 2.3 μM to 150 μM, and analyzed by CD spectroscopy. The GFOGER dilutions (Fig. 2D) show CD spectra characteristics of a triple helix in the range of 9.4–150 μM, while the UPy-GFOGER dilutions (Fig. 2E) show CD spectra characteristics of a triple helix in the range of 18.8–150 μM. These results suggest a slightly enhanced stabilization of the triple helix formation for the GFOGER polypeptide compared with the conjugated UPy-GFOGER. For future studies it would be beneficial to study the longevity of the triple helical structures under physiological conditions.

### 2.4 The impact of mixing UPy-Glycine and UPy-GFOGER on triple helix formation

**Fig. 3:**
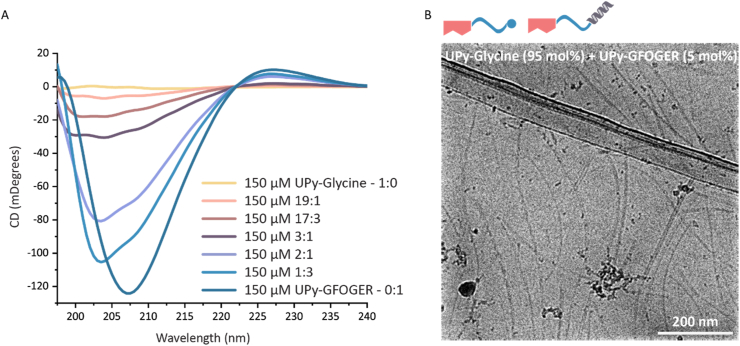
Conformational change of UPy-GFOGER upon mixing and dilution by UPy-Glycine. **A)** CD spectra of UPy-GFOGER and UPy-Glycine mixtures at various concentration ratios at 37 °C. **B)** Cryogenic transmission electron micrograph of supra molecular assemblies of UPy-GFOGER and UPy-Glycine at a 19:1 mol ratio (orange graph in CD spectra). (For interpretation of the references to colour in this figure legend, the reader is referred to the Web version of this article.)

To allow the incorporation of the supramolecular collagen mimicking UPy-GFOGER additive into a synthetic UPy-based hydrogel it was necessary to mix UPy-Glycine with UPy-GFOGER. For this purpose, it was considered to be interesting to study the effect on triple helix formation by dilution of the GFOGER segment. To this end, stock solutions were prepared in alkaline PBS buffer both at a concentration of 150 μM of UPy-GFOGER and 150 μM of UPy-Glycine. The stock solutions were mixed at desired ratios, neutralized with HCl solution, and allowed to equilibrate overnight. Except for the pure UPy-Glycine, all the mixtures showed a CD effect (Fig. 3A). The CD spectra of the mixtures showed a decreased CD single upon dilution of the GFOGER segment by an increased addition of UPy-Glycine. At an UPy-GFOGER mol content of less than a third, the characteristic CD spectrum, which refers to a triple helix, was lost. Yet, the CD spectra of the mixtures with even lower content of UPy-GFOGER still showed optical activity. To this end, a mixture with only 5 mol% UPy-GFOGER (referring to the 19:1 mixture) was visualized through cryo-TEM to investigate the morphologies of the supramolecular assemblies. Long nanofibers were observed within the mixture via the obtained cryo-TEM images. These nanofibers lacked the brush-like morphology, which was assumed to be characteristic for the UPy-GFOGER (Fig. 3B). Taken together, dilution by addition of UPy-Glycine resulted in a change of the polypeptide segment in UPy-GFOGER, from a triple helix into a random coil conformation. Furthermore, these results indicated that the assembly behavior is mainly dominated by the dimerizing UPy-moieties instead of by the polypeptide GFOGER segment.

### 2.5 3D culture of human primary keratocytes encapsulated in synthetic collagen mimicking hydrogels

**Fig. 4:**
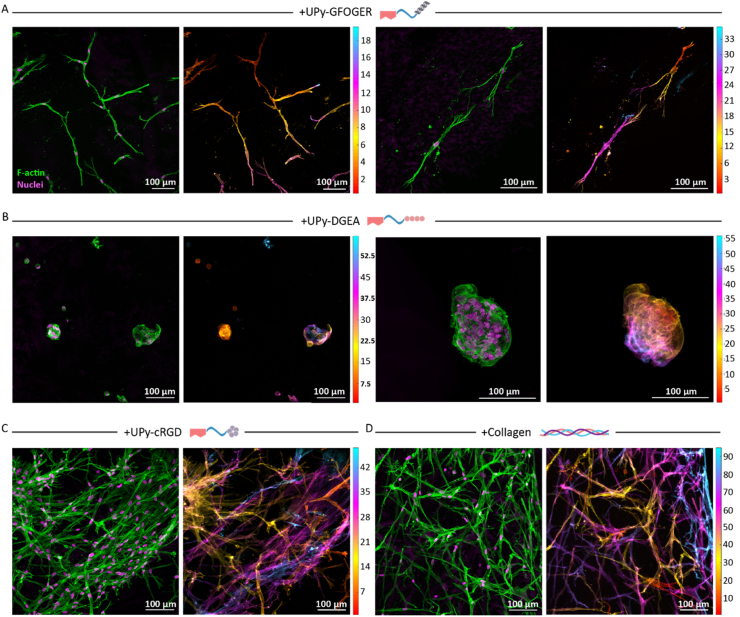
UPy-GFOGER and UPy-DGEA functionalized hydrogels allow for 3D encapsulation and culture of PKs. For all the images accounts the following: F-actin is in green, nuclei are in magenta and the scale bars represent 100 μm. The scale of the height images is in μm. **A)** The cellular results of the synthetic UPy-GFOGER. **B)** The cellular results of the synthetic UPy-DGEA. **C)** The cellular results of the synthetic ‘golden standard’ control UPy-cRGD. **D)** The cellular results of the hybrid collagen type I hydrogel. (For interpretation of the references to colour in this figure legend, the reader is referred to the Web version of this article.)

After successful synthesis of the collagen mimicking UPy-GFOGER and a thorough characterization of the conformational properties of UPy-GFOGER, both the UPy-GFOGER and the UPy-DGEA additive were used to generate full synthetic hydrogels, allowing for 3D cell culture of primary keratocytes. Donor derived human primary keratocytes (PK) were encapsulated within hydrogels functionalized with either the UPy-GFOGER or the UPy-DGEA and cultured up to 21-days. The + UPy-GFOGER functionalized hydrogel supported the PKs to attain their healthy elongated morphology (Fig. 4A). The + UPy-DGEA functionalized hydrogel was not able to support the healthy morphology of the encapsulated PKs, resulting in clusters of round-shaped cells (Fig. 4B). Both the synthetic hydrogel functionalized with UPy-cRGD as well as the hybrid hydrogel mixed with natural collagen type I were used as reference hydrogels [51]. Previous work demonstrated only minor differences in mechanical characteristics as a result of the incorporation of an UPy peptide additive, therefore no additional rheological measurements were performed in this study (SI, Fig. S2). Additionally, a small comparative study with a fully natural collagen hydrogel was conducted to evaluate the performance of the synthetic and hybrid hydrogels (SI, Fig. S3 [61]).

**Fig. 5:**
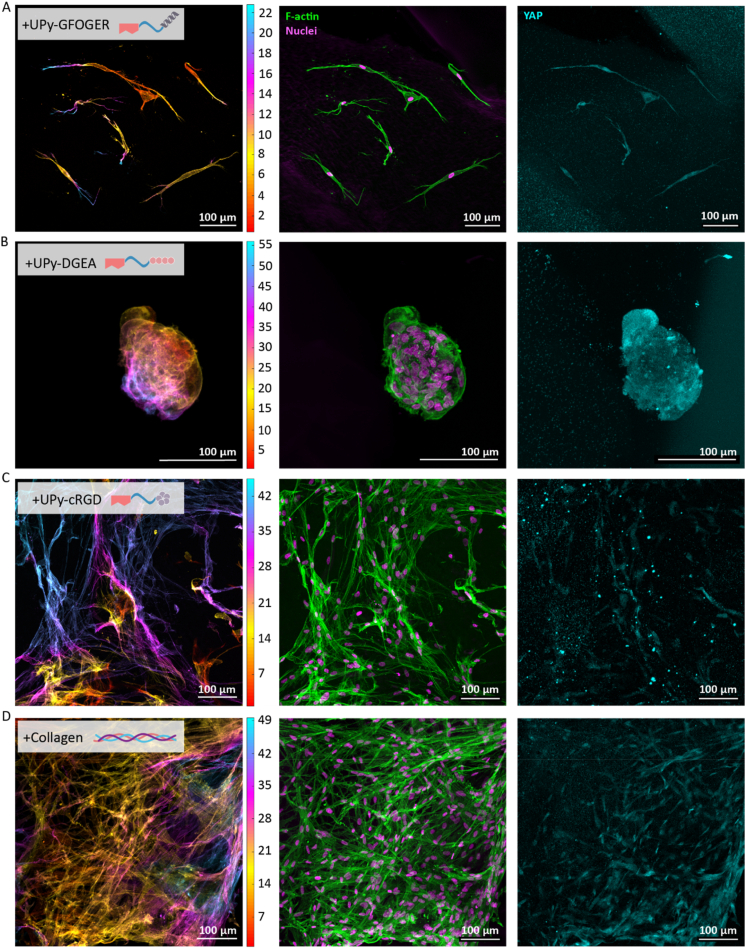
Encapsulated PKs sensed similar mechanotransduction when encapsulated in the synthetic + UPy-GFOGER and +UPy-DGEA hydrogels, compared with the +UPy-cRGD and hybrid hydrogel control when cultured for 21 days. Height images are in μm scale, F-actin is in green, nuclei are in magenta and YAP is in cyan. Scale bars represent 100 μm. **A)** The results of the synthetic + UPy-GFOGER hydrogel. **B)** The results of the synthetic + UPy-DGEA hydrogel. **C)** The results of the synthetic + UPy-cRGD hydrogel. **D)** The results of the hybrid + collagen hydrogel. (For interpretation of the references to colour in this figure legend, the reader is referred to the Web version of this article.)

Multicolored images show the various heights of the cells within the z-direction of the hydrogel, demonstrating the ability of the cells to elongate and grow in the provided 3D environment. The results of the +UPy-cRGD (Fig. 4C) and +Collagen (Fig. 4D) control showed a high density of elongated cells, similar to the previous results of these two hydrogel conditions. Together, these results demonstrated a novel synthetic hydrogel functionalized with UPy-GFOGER, which supports PKs to adhere and to adapt to their healthy elongated morphology in a similar fashion as the +UPy-cRGD and +Collagen functionalized hydrogels. The + UPy-DGEA hydrogel showed to be less suitable for the encapsulation of keratocytes, yet this hydrogel could be useful for cell types with a preference for cell-cell interactions above cell-matrix interactions, *i.e.* organoids. Mechanotransduction allows cells to sense and adapt to external forces by cytoskeleton and focal adhesion (FA) remodeling or activating specific genetic programs [62]. To explore the interaction between the cells and the material in more detail, an analysis of yes-associated-protein (YAP) was implemented in this study. The immunohistochemical images of PKs encapsulated and cultured in the various hydrogels showed the expression of YAP within the cytoplasm and/or nuclei. For the +UPy-GFOGER (Fig. 5A), the +UPy-DGEA hydrogel (Fig. 5B) and the +UPy-cRGD hydrogel (Fig. 5C) it was difficult to observe a difference in YAP expression between the cytoplasm and the nuclei. On the contrary, nuclear YAP seemed to be expressed in the majority of the PKs encapsulated within the hybrid hydrogel (+Collagen) (Fig. 5D). These variations could be explained by the difference in cell density (SI, Fig. S4). Compared with the sparse elongated cells present within the +UPy-GFOGER hydrogel, the hybrid hydrogels demonstrated a more dense cell network, favoring cell-cell interactions that affect the activation and expression of YAP within the nuclei [63].

Continuation of the UPy-DGEA and UPy-GFOGER additives in future work demands more detailed cell studies, for instance analyzing the influence of the additives on cell proliferation or analyzing the influence of the additives on the expression and activation of receptors on the keratocytes.

### 2.6 Differentiation of primary keratocytes encapsulated in synthetic collagen mimicking hydrogels

After a successful encapsulation of the PKs within the hydrogels functionalized with collagen mimicking peptides, the PKs were treated in two manners; (1) with serum towards stromal fibroblasts (SFs) or (2) with low concentration of serum and high concentration of glucose towards quiescent corneal stromal keratocytes (CSKs) [64]. At first all the hydrogels were embedded in medium with serum to allow adherence of the PKs to the material. On culture day 1, the medium is changed to low serum and high glucose medium, allowing the PKs to (re-)differentiate towards CSKs during the 21-day culture.

**Fig. 6:**
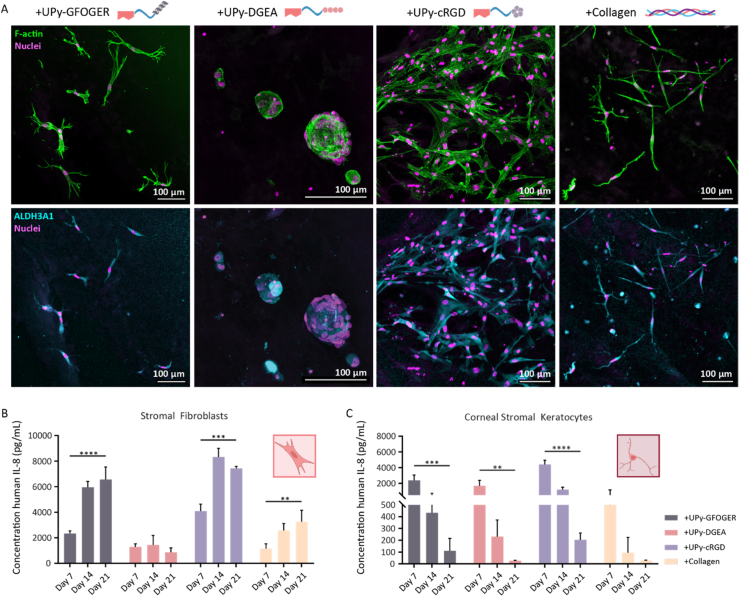
PKs (re)differentiated towards stromal fibroblasts and corneal stromal keratocytes when encapsulated and cultured in the synthetic hydrogels functionalized with either UPy-GFOGER or UPy-DGEA, n = 3. **A)** Immuno fluorescent staining of F-actin (green), nuclei (magenta) and ALDH3A1 (cyan). Scale bar represents 100 μm. **B)** Interleukin 8 (IL-8) secretion on culture day 7, 14, and 21 by PKs encapsulated within the various hydrogels and treated towards stromal fibroblasts. Sample size n = 3, **p < 0.01, ***p < 0.001, ****p < 0.0001, mean ± standard deviation presented. **C)** Interleukin 8 (IL-8) secretion on culture day 7, 14, and 21 by PKs encapsulated within the various hydrogels and treated towards corneal stromal keratocytes. Sample size n = 3, **p < 0.01, ***p < 0.001, ****p < 0.0001, mean ± standard deviation presented. (For interpretation of the references to colour in this figure legend, the reader is referred to the Web version of this article.)

Healthy human keratocytes do express crystallin proteins, which reduce light scattering and enhance the optical performance of the stromal tissue. One of these proteins is the enzyme aldehyde dehydrogenase 3 Family member A1 (ALDH3A1), which is often used as a specific marker for keratocytes [65,66]. Here, immunohistochemical staining showed the expression of ALDH3A1 in PKs treated towards CSKs (Fig. 6A). Within the cell clusters formed by the PKs encapsulated and cultured within the hydrogel functionalized with UPy-DGEA, a less clear ALDH3A1 expression was observed. In contrast, the PKs encapsulated and cultured within the hydrogels functionalized with UPy-GFOGER, UPy-cRGD and collagen all showed a distinct ALDH3A1 expression, indicating that the PKs start to comprise specific keratocyte behavior. During this 21-day cell culture, small amounts of cell culture medium were stored to perform an enzyme-linked-immunosorbent assay (ELISA). From literature it is known that SFs secrete among others the cytokine Interleukin-8 (IL-8), which is a pro-inflammatory molecule responsible for neutrophil recruitment and T-cell activation. Since, CSKs barely secrete the cytokine IL-8 an ELISA based on IL-8 secretion was used to analyze the cellular (re-)differentiation possibilities of PKs encapsulated and cultured within hydrogels functionalized with collagen mimicking peptides [67]. PKs treated with high serum towards SFs and encapsulated within the +UPy-GFOGER, +UPy-DGEA, +UP-cRGD, and +collagen hydrogel secreted; 6562 ± 979 pg/mL, 867 ± 349 pg/mL, 7429 ± 160 pg/mL, and 3262 ± 889 pg/mL IL-8, respectively (Fig. 6B). While, PKs treated with low serum and high glucose towards CSKs encapsulated within the +UPy-GFOGER, +UPy-DGEA, +UP-cRGD, and +collagen hydrogel secreted; 111 ± 105 pg/mL, 27 ± 4 pg/mL, 204 ± 58 pg/mL, and 29 ± 2 pg/mL IL-8, respectively (Fig. 6C). Significant differences were observed between the secreted IL-8 by PKs treated towards SFs or CSKs, while being encapsulated within the UPy-GFOGER functionalized hydrogel. These results also corresponded with the results of the two controls. Solely, the secreted IL-8 concentration by PKs treated towards SFs or CSKs while encapsulated within the hydrogel functionalized with UPy-DGEA, showed no significant differences. Furthermore, the results demonstrated a significant increase in secreted IL-8 concentration for the PKs treated towards SFs between day 7 and day 21. On the contrary, the results demonstrated a significant decrease in secreted IL-8 concentration for the PKs treated towards CSKs between day 7 and day 21. The increase in IL-8 concentration for the PKs treated towards SFs and the decrease in IL-8 concentration for the PKs treated towards CSK accounts for all the hydrogels, except the +UPy-DGEA functionalized hydrogel. Taken together, both the increase in ADLH3A1 expression as well as the significant decrease of secreted IL-8, demonstrated that the hydrogel functionalized with UPy-GFOGER allows the PKs to (re-)differentiate into CSKs.

## 3 Conclusion

Here, both UPy-GFOGER and UPy-DGEA are introduced as two supramolecular modified collagen type I mimicking peptides. Both CD spectroscopy as well as cryo-TEM demonstrated the self-assembly behavior with respect to the triple-helical formation of UPy-GFOGER, resulting in a novel superstructure. Encapsulation and culture of PKs within a hydrogel functionalized with UPy-GFOGER or UPy-DGEA demonstrated distinct differences in cellular behavior. Round-shaped cells and cell clumps were observed for the PKs cultured within the UPy-DGEA functionalized hydrogel, while the PKs cultured in UPy-GFOGER functionalized hydrogels attained an elongated morphology. Moreover, PKs encapsulated and cultured within UPy-GFOGER functionalized hydrogels (re-)differentiate into CSKs.

While the synthesis of UPy-DGEA is easier and faster compared with the synthesis of UPy-GFOGER, the induced bioactivity of the short additive is not sufficient. Herewith, we demonstrated the need of a collagen mimicking peptide additive with a complex structure to provide the desired biochemical cues for the cells. With this novel UPy-GFOGER molecule we designed a collagen type I mimicking bioactive additive, which allows the formation of a synthetic stromal microenvironment, mimicking closely the natural ECM.

## 4 Experimental section

### 4.1 Synthesis of GGG-GPP5-GFOGER-GPP5 (GFOGER) peptide

Standard Fmoc solid-phase peptide synthesis on a rink resin at 0.150 mmol scale was used to synthesize the Gly-Gly-Gly-(Gly-Pro-Pro)_5_-Gly-Phe-Hyp-Gly-Glu-Arg-(Gly-Pro-Pro)_5_ polypeptide. The F-moc group was removed from Fmoc-Gly-Gly-Gly-(Gly-Pro-Pro)_5_-Gly-Phe-Hyp(*t*Bu)-Gly-Glu(OtBu)-Arg-(Gly-Pro-Pro)_5_ (567 mg, 0.3 mmol) using 20 v/v% piperidine in DMF for 2 × 15 min, while still on the rink amide resin. A cleaving mixture of 95:2.5:2.5 v/v% TFA:TIS:H_2_O for 2 h was used to cleave the peptide from the resin. To yield a whit fluffy solid, the cleavage mixture was precipitated 5x in ice-cold Et_2_O and subsequently freeze-dried. Preparative reverse phase LC-MS on a C18 column using a gradient of 20–25% of acetonitrile in H_2_O, containing 0.1% TFA was used to purify the crude compound. After purification, the final yield was 107 mg GFOGER peptide (19%), which had a purity of >95%.

#### 4.1.1 Synthesis of UPy-GFOGER

The GFOGER polypeptide (84 mg; 0.025 mmol) was dissolved in 4 mL DMF. The UPy-synthon was synthesized as previously reported [68]. The UPy-synthon (37 mg; 0.32 mmol) was dissolved in 3 mL DMF. DIPEA (26 μL; 0.15 mmol) and HARU (13.3 mg; 0.035 mmol) were dissolved in 2 mL DMF and added to the UPy-synthon solution, whereafter the complete mixture was stirred for 15 min at room temperature. After pre-activation the reaction mixture was added to the GFOGER polypeptide solution and reacted overnight under shaking conditions at room temperature. To yield a white fluffy solid, the cleavage mixture was precipitated 5x in ice-cold Et_2_O and subsequently freeze-dried. Preparative reverse phase LC-MS on a C18 column using a gradient of 30–35% of acetonitrile in H_2_O, containing 0.1% TFA was used to purify the crude compound (SI, Fig. S1). After purification, the final yield was 16 mg UPy-GFOGER peptide (13%), which had a purity of >5%.

#### 4.1.2 Synthesis of UPy-GCGDGEA peptide additive via maleimide-thiol conjugation

The Gly-Cys-Gly-Asp-Gly-Glu-Ala peptide was synthesized using standard Fmoc solid-phase peptide synthesis on a rink amide resin at 0.1 mmol scale. The Fmoc-group was removed using 20 v/v% piperidine in DMF for 2 × 15 min, while still on the rink amide resin. Subsequently, the peptide was cleaved from the resin in a 90:2.5:2.5:5 v/v% TFA:TIS:H_2_O:EDT for 2 h. The cleavage mixture was precipitated 6x in ice-cold Et_2_O. Preparative reverse phase LC-MS on a C18 column using a gradient of 20–25% of acetonitrile in H_2_O, containing 0.1% TFA was used to purify the crude compound. The GCGDGEA peptide was attached to the UPy-moiety via maleimide-thiol chemistry, where Tris(2-carboxyethyl)phosphine (TCEP) is used to cleave the sulfide bonds and therewith activate the thiol in the cysteine to react with the maleimide (SI, Fig. S1).

#### 4.1.3 Circular dichroism spectroscopy

Stock solutions (300 μM) were prepared by dissolving GFOGER, UPy-GFOGER, and UPy-Glycine in alkaline pBS solutions (containing 80 mM NaOH in PBS) and heated to 70 °C for 1 h. For the preparations of the mixtures the stock solutions were diluted 2x in alkaline PBS solution and mixed in the desired ratios, followed by neutralization by using 1 M HCl. Afterwards the samples were left overnight at 4 °C to allow assembly. Samples were measured at a scan speed of 100 nm per min, with a data pitch of 0.25 nm, a response time of 0.5 s, and a bandwidth of 2 nm. A suprasil® quartz cuvette was used with a pathlength of 1 mm, and a chamber volume of 350 μL (Hellma Analytics). The spectra were measured from 400 to 170 nm at 37 °C. Graphs are shown from 197.5 to 240 nm, due to noise observed below 200 nm due to wavelength absorption of water.

#### 4.1.4 Nile red assay

150 μM stock solutions with a volume of 1 mL were prepared by dissolving GFOGER, UPy-GFOGER, and UPy-Glycine at 70 °C in alkaline PBS solutions (containing 80 mM of NaOH) for 1 h. After cooling down to room temperature, 1.5 μL of 5 mM Nile red stock was added to each solution, and the solutions were neutralized with 1 M HCl. The solutions were left overnight at 4 °C to equilibrate before the measurements were performed. A Varian Fluorescence spectrophotometer was used to measure the Nile red assays. After excitation of the samples the fluorescence emission scans were recorded from 560 to 800 nm at a fixed temperature of 37 °C. the excitation and emission slits were fixed at 5 nm, and the scan rate used was 600 nm per min, PMT detector voltage was set to 1000 V. Both the sample preparation for the Nile red assay as well as the actual measurement was performed in collaboration with Johnick F. van Sprang.

#### 4.1.5 Cryogenic transmission electron microscopy

150 μM stock solutions were prepared by dissolving GFOGER, UPy-GFOGER, and UPy-Glycine at 70 °C in alkaline PBS solutions (containing 80 mM of NaOH) for 1 h. After cooling down, the desired mixtures were made and the, if necessary, and then the solutions were mixed thoroughly and neutralized with 1 M HCl. Lacey carbon film grids (200 mesh, 50 μm hole size; Electron Microscopy Sciences) were surface plasma treated at 5 mA for 40 s using a Cressington 208 carbon coater, and each dispersion (3 μL) was applied onto each grid. Using an automated vitrification robot (FEI Vitrobot Mark III), excess sample was removed through blotting with filter paper for 3 s at −3 mm. Thin films of dipsersions were vitrified by plunging the grids into liquid ethane, which is just above its freezing point. A FEI-Titan TEM equipped with a field emission gun operating at 300 kV was used to carry out imaging. Samples were imaged using a post-column Gatan energy filter and a 2048x2048 Gatan CCD camera. Micrographs were recorded at low dose conditions, using a defocus setting of −10 μm at 25.000× magnification.

#### 4.1.6 Primary keratocyte (PK) cell culture

Primary human keratocytes were isolated from leftover human corneoscleral transplant material from Descemet Membrane Endothelial Keratoplasty surgery, which were obtained from the Cornea Department of the ETB-BISLIFE Multi-Tissue Center (Beverwijk, the Netherlands). The keratocytes were cultured in expansion medium (1:1 mixture of Dulbecco's modified Eagle's medium/F-12 supplemented with GlutaMAX (DMEM/F12 (Ham) + GlutaMAXTM, 10565–018; Gibco), 5% Fetal Bovine Serum (FBS, Biochrom AG), 1% penicillin/streptomycin (P/S, Biochrom AG), and 1 mM l-ascorbic acid 2-phosphate sesquimagnesium salt hydrate (Vitamin C, Sigma A8960)) at 37 °C, 21% O_2_ and 5% CO_2_ until ± 80% confluency. Since the medium contained FBS, keratocytes were considered to be activated matrix-producing cells, referred to as stromal fibroblasts (SFs). To initiate cell (re-)differentiation towards corneal stromal keratocytes, another medium composition was used: differentiation medium (Dulbecco's modified Eagle's medium supplemented with GlutaMAX (GlutaMAXTM, 11880–028; Gibco), 1% penicillin/streptomycin (P/S, Biochrom AG), and 1 mM l-ascorbic acid 2-phosphate sesquimagnesium salt hydrate (Vitamin C, Sigma A8960), 1x ITS (Sigma, I3146), 2 mg/mL d-glucose (Invitrogen, 15023021), 2.5 mg/mL D-mannitol (Fluka, 63560)) [64]. Cells were cultured with medium changes every 3 days, keratocytes from multiple donors were used up to passage #3. TrypLE Express Enzyme (1x), no phenol red (12604013, Gibco) is used to detach the cells from the culture flask and use them for experiments.

#### 4.1.7 Cell encapsulation in hydrogels

Both the bifunctional as well as the monofunctional building blocks were received as powders. The bifunctional molecules (UPy-PEG_10K_-UPy) were dissolved at 70 °C in a neutral PBS solution for 1.5 h. Monofunctional molecules (UPy-Glycine + UPy-cRGD or UPy-Glycine + UPy-GFOGER or UPy-Glycine + UPy-DGEA) were dissolved at 70 °C in an alkaline PBS solution (containing 160 mM NaOH) for 20 min. After completely dissolving the powders of the bifunctional and monofunctional building blocks, the solutions were cooled down to room temperature. A specific volume of HCl solution (2 M) was added to the solution of monofunctional molecules to reach a neutral pH. Cell culture medium is added to both solutions 1:1, to provide the cells already with some nutrients during the gelation process later on in the procedure. Afterwards, both solutions are transferred from a glass vial to a sterile Eppendorf tube, from this step onwards a safety cabinet is used to guarantee a sterile work environment. The solutions were disinfected by exposing them to UV-light for 20 min. Subsequently, the cells were prepared and counted, the following cell concentrations were used during the experiments:

HCKs: 100 cells/mL Primary keratocytes treated towards SFs: 100 cells/μL Primary keratocytes treated towards CSKs: 200 cells/μL.

The cells needed for encapsulation were suspended in the correct amount of medium and this cell suspension was added to the solution of bifunctional molecules. Due to the addition of the cells suspension, the bifunctional molecules were diluted. To correct for this extra dilution step, the initial concentration of the bifunctional molecules was slightly higher (0.78 wt/v% instead of 0.52 wt/v%). For every experiment the bifunctional molecules were diluted with 1/3 of cell suspension, resulting in a final 0.52 wt/v% of bifunctional molecules. All the gels were prepared in wells of a non-adhesive 96-well plate (Fisher Scientific, Nunclon Sphera-Treated, U-Shaped-Bottom plate 15227905).

#### 4.1.8 Synthetic hydrogels

First 40 μL monofunctional molecules in solution were added to a well. Meanwhile, the cells were added to the solution of bifunctional molecules and mixed thoroughly. Second, 40 μL of bifunctional molecules/cell mixture were added to the 40 μL solution of monofunctional molecules inside the well. The molecules were mixed by carefully pipetting up and down (at least 3x per well), any air bubbles were removed by using a needle.

#### 4.1.9 Hybrid hydrogel (control hydrogel)

For hybrid hydrogel preparation collagen type I (Gibco, Collagen I bovine, A1064401) was added to the solution of the bifunctional molecules, 20 μL was added per 100 μL complete gel (0.1 wt/v%). Initially, the bifunctional molecules were dissolved at a higher wt/v% compared with the bifunctional molecules used for the synthetic or pristine hydrogel, to correct for this extra dilution step with the collagen. At first, 40 μL solution of monofunctional molecules was added to a well. The cells and the collagen were added to the solution of bifunctional molecules and mixed thoroughly. Second, 40 μL of bifunctional molecules/cell/collagen mixture were added to the 40 μL solution of monofunctional molecules inside the well. The molecules were mixed by carefully pipetting up and down (at least 3x per well), any air bubbles were removed by using a needle.

For the exact hydrogel compositions, see Table 1. All the hydrogels were placed up-side down in the incubator at 37 °C, 21% O_2_ and 5% CO_2_ for 1 h to allow for proper gelation. After 1 h, medium to embed the gels was carefully added to the wells, and the hydrogels with encapsulated cells were placed back in the incubator at 37 °C, 21% O_2_ and 5% CO_2_. After 1 day, the cell culture medium, surrounding the hydrogel is refreshed and for the PKs treated towards CSKs the expansion medium is replaced by the differentiation medium.

**Table 1:** Overview of the four hydrogel compositions used within this study. Calculations were made for one complete hydrogel with a volume of 80 μL.

Hydrogel compositions					
Synthetic hydrogel (+UPy-cRGD)					
Building block/additive	Ratio	μmol	mM	Wt/v %	Total Wt/v %
UPy-PEG_10K_-UPy	1	0.0094	0.117	0.13	1.29
UPy-Glycine	73.6	0.69	8.63	1.03	
Additive	6.4	0.06	0.75	0.13	
**Synthetic hydrogel (**+**UPy-GFOGER)**					
Building block/additive	Ratio	μmol	mM	Wt/v %	Total Wt/v %
UPy-PEG_10K_-UPy	1	0.0094	0.12	0.13	1.42
UPy-Glycine	75.6	0.71	8.85	1.06	
Additive	4.4	0.04	0.52	0.23	
**Synthetic hydrogel (**+**UPy-DGEA)**					
Building block/additive	Ratio	μmol	mM	Wt/v %	Total Wt/v %
UPy-PEG_10K_-UPy	1	0.0094	0.12	0.13	1.3
UPy-Glycine	73.6	0.69	8.63	1.03	
Additive	6.4	0.06	0.75	0.14	
**Hybrid hydrogel (**+**collagen)**					
Building block/additive	Ratio	μmol	mM	Wt/v %	Total Wt/v %
UPy-PEG_10K_-UPy	1	0.0094	0.12	0.13	1.25
UPy-Glycine	83.3	0.75	9.38	1.12	
Collagen	0.1 wt/v%				

#### 4.1.10 Cell staining and imaging

Before immunohistochemical stainings were carried out, the hydrogels were washed 3x with PBS (5 min per wash). All cells encapsulated within the hydrogels were fixated for 20 min at room temperature using 3.7% paraformaldehyde (formalin 37%, 104033.1000, Merck). After washing with PBS, samples were permeabilized for 15 min with 0.5% Triton X-100 in PBS. Followed by adding a blocking solution of 10% donkey or goat serum in 0.05% Triton X-100 in PBS for 30 min. Next, the cells were incubated with the primary antibodies diluted in 2% goat serum in 0.05% Triton X-100 in PBS overnight at 4 °C. Thereafter, the cells were washed thoroughly with 0.05% Triton X-100 in PBS, including wash waiting steps of 5–10 min. Next, the cells were incubated with the secondary antibodies and phalloidin at room temperature for 2 h. Finally, the cells were stained with DAPI at a dilution of 1:250 for 10 min and washed thoroughly with PBS (including was waiting steps of 5–10 min). During imaging, complete/intact hydrogels were place on a thin coverslip (24 × 69 mm, VWR 631–1575) immerged in mowiol 4–88 (Sigma Aldrich, 81381) and imaged using a Leica TCS SP8 X inverted confocal microscope (Leica Microsystems) using HC PL APO CS2 objectives (20×/0.75, 40x/0.95). Images were processed in ImageJ to create a max-projection image of the original z-stack. See Table 2 for the primary and secondary antibodies used within this study.

**Table 2:** Overview of the used dyes, primary-, and secondary antibodies.

Antibody/dye	Company/reference #	Dilution
**4′,6-diamidino-2-phenylindole dihydrochloride (DAPI)**	Sigma-Aldrich, D9542	1:250
**Phalloidin 488**	Sigma-Aldrich	1:300
**Phalloidin 555**	Sigma-Aldrich	1:300
**Anti-YAP1**	Ab52771, Abcam	1:100
**Anti-ALDH3A1**	Ab76976, Abcam	1:100
***Secondary antibody***		
**Anti-rabbit IgG (goat) 647**	A21244, Molecular Probes	1:250

#### 4.1.11 Enzyme-linked immunosorbent assay (ELISA)

On various days during the 3D cell culture, medium was refreshed and all medium surrounding the sample was collected in separate small Eppendorf tubes and stored in −80 °C upon use. All ELISA experiments were executed with n = 3, and all the standards as well as the samples are ran in duplicate. The following IL-8 concentrations were used for the standard curve of IL-8: 1000 pg/mL, 500 pg/mL, 250 pg/mL, 125 pg/mL, 62.5 pg/mL, 31.3 pg/mL, 15.6 pg/mL.

Uncoated Nunc™MaxiSorp™ ELISA plates (BioLegend, 423501) were used, and coated with capture antibody diluted in 1x coating buffer one day prior to running the ELISA (overnight incubation at 4 °C). See Table 3 for all the compositions of the used reagents. On the experiment day all the samples, the plates and the ELISA MAX™ Deluxe Set components (BioLegend®) were brought to room temperature. All the wash steps were executed in a similar manner, namely: 4 times with at least 300 μL wash buffer (0.05% Tween-20 in PBS) per well and residual buffer was blotted by firmly tapping the plate upside down on absorbent paper. After incubation with the capture antibody the plate was washed and blocked with 1x assay diluent A at room temperature for 1 h with shaking (500 rpm) to minimize non-specific binding and reduce background. During this incubation, the samples for the standard curve were prepared and every well plate comprised a standard. After blocking, the wells were washed and incubated with 100 μL of the samples and standards at room temperature for 2 h with shaking (500 rpm), all samples were diluted 4x in 1x assay diluent (25 μL sample, 75 μL 1x assay diluent). Thereafter, the wells were washed and 100 μL of detection antibody solution was added to each well, and incubated at room temperature for 1 h with shaking (500 rpm). The wells were washed again and incubated with 100 μL of diluted Avidin-HRP solution at room temperature for 30 min with shaking (500 rpm). Subsequently, the wells were washed 5x thoroughly with wash buffer, and soaked in buffer for 1 min for each wash to minimize background. Then, 100 μL of substrate solution C was added and incubated in the dark for 15 min. During this step, positive wells turned blue in color. To stop the reaction, 100 μL Stop Solution for TMB Substrate (BioLegend, 4230021) was added to each well. All the positive wells turned from blue to yellow during this step. After adding the stop solution to the wells, the plate was softly tapped to the table a few times to proper mix the solutions and immediately the absorbance at 450 nm was measured with the plate reader (Synergy HTX, BioTek). In addition the absorbance at 570 nm was measured, this absorbance could be subtracted from the 450 nm absorbance. The standard curve for each ELISA was plotted in excel and polynomial curve-fitting software with order 3 or 4 was used to determine the best fit. Using this formula together with the measured absorbance value, resulted in the calculation of the amount of cytokine secretion. Graphpad Prism was used to perform statistical analysis. A 2-way Anova combined with a Sídák's multiple comparisons test demonstrated significant differences between the cellular IL-8 secretion of cells treated as stromal fibroblasts or cells treated towards corneal stromal keratocytes between day 7 and 21 for all the hydrogels, except the hydrogel functionalized with UPy-DGEA.

**Table 3:** Reagents used to perform an ELISA with 2x a complete 96-well plate.

4 mL Coating Buffer A (5x)	16 mL of Deionized Water
**100 μL of Capture Antibody (200x)**	20 mL of 1x Coating Buffer
**12 mL of Assay Diluent A (5x)**	48 mL of PBS
**100 μL of Detection Antibody (200x)**	20 mL of 1x Assay Diluent A
**20 μL of Avidin-HRP (1000x)**	20 mL of 1x Assay Diluent A
**Substrate Solution C**	–

Used ELSIA set: *ELISA MAX™ Deluxe Set Human IL-8 (BioLegend, 431504)*.

#### 4.1.12 3D height indication cell images

A custom MatLab script was written to pair a specific height of the cell within the obtained z-stack image to a specific color. Image stacks obtained from channels corresponding to the DAPI (nuclei) and phalloidin (F-actin) were used to create these 3D height indication cell images. LAS X Life Science Microscope Software was used to calculate the original μm distance in the z-direction from the original *lif* file. *The custom MatLab script was written by Mark C. van Turnhout.*

## CRediT authorship contribution statement

**Annika F. Vrehen:** Writing – original draft, Methodology, Investigation, Data curation, Conceptualization. **Johnick F. van Sprang:** Methodology, Investigation, Data curation. **Maaike J.G. Schotman:** Investigation, Data curation. **Patricia Y.W. Dankers:** Writing – review & editing, Supervision, Resources, Project administration, Methodology, Funding acquisition, Conceptualization.

## Declaration of competing interest

The authors declare the following financial interests/personal relationships which may be considered as potential competing interests: Patricia Dankers reports financial support was provided by 10.13039/501100003005Eindhoven University of Technology. Patricia Dankers reports a relationship with Eindhoven University of Technology that includes: employment. Patricia Dankers is co-founder and share holder of UPyTher and VivArt-X (two spin-off companies from the group). If there are other authors, they declare that they have no known competing financial interests or personal relationships that could have appeared to influence the work reported in this paper.

## Data Availability

Data will be made available on request.
