# Supplementary material for: Collagen type I mimicking peptide additives to functionalize synthetic supramolecular hydrogels
Source: Mater Today Bio. 2024 Mar 15;26:101021. doi: 10.1016/j.mtbio.2024.101021 (PMC10966780; doi:10.1016/j.mtbio.2024.101021)

**Supporting Information**

**Collagen type I mimicking peptide additives to functionalize synthetic supramolecular hydrogels**

*Annika F. Vrehen, Johnick F. van Sprang, Maaike J. G. Schotman, Patricia Y. W. Dankers**


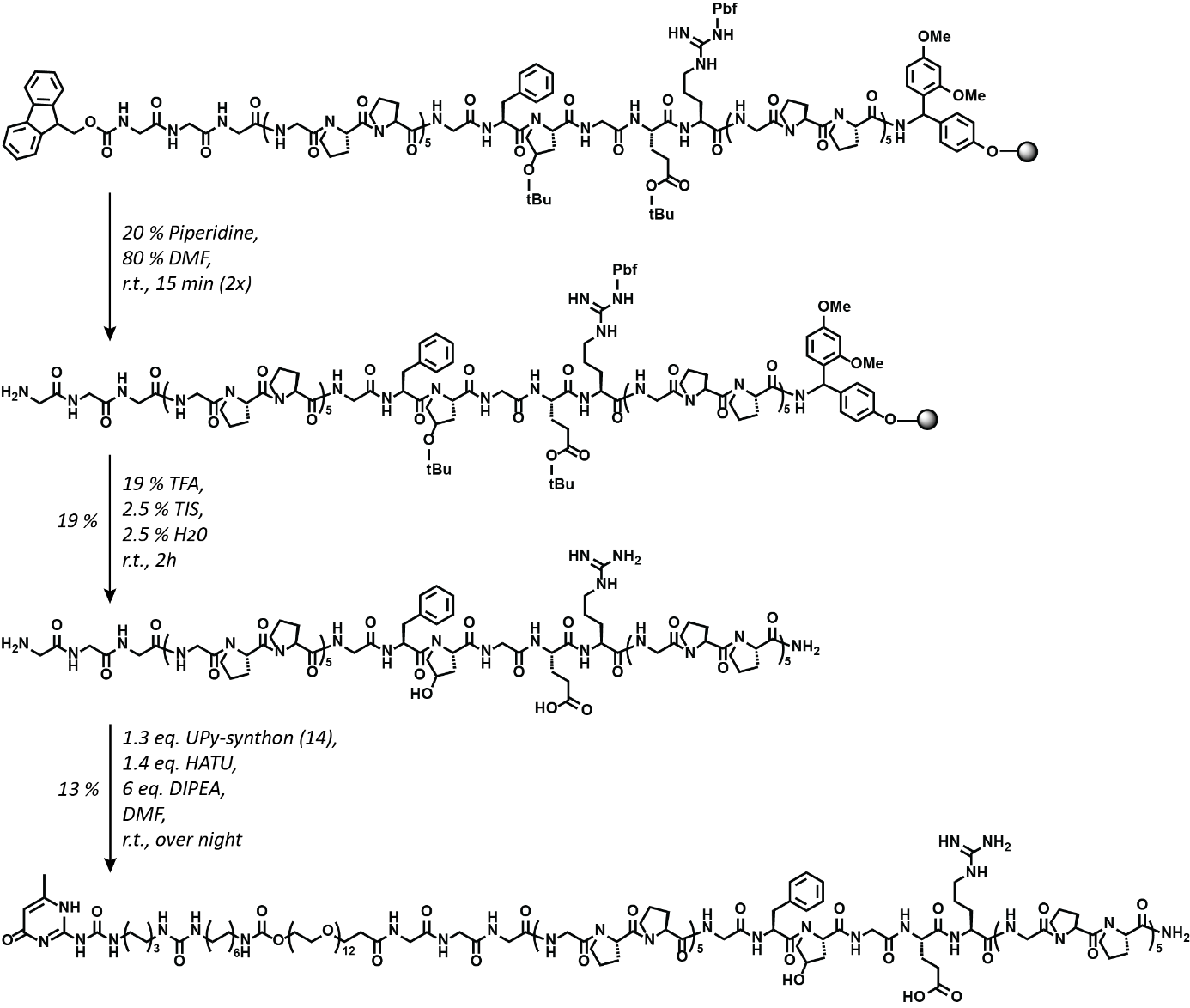


**Scheme S1** Synthesis of GGG-GPP_5_-GFOGER-GPP_5_ polypeptide and the conjugation to a UPy-moiety via the N-terminus of the polypeptide, resulting in the supramolecular bioactive additive UPy-GGG-GPP_5_-GFOGER-GPP_5_ (referred to as UPy-GFOGER).


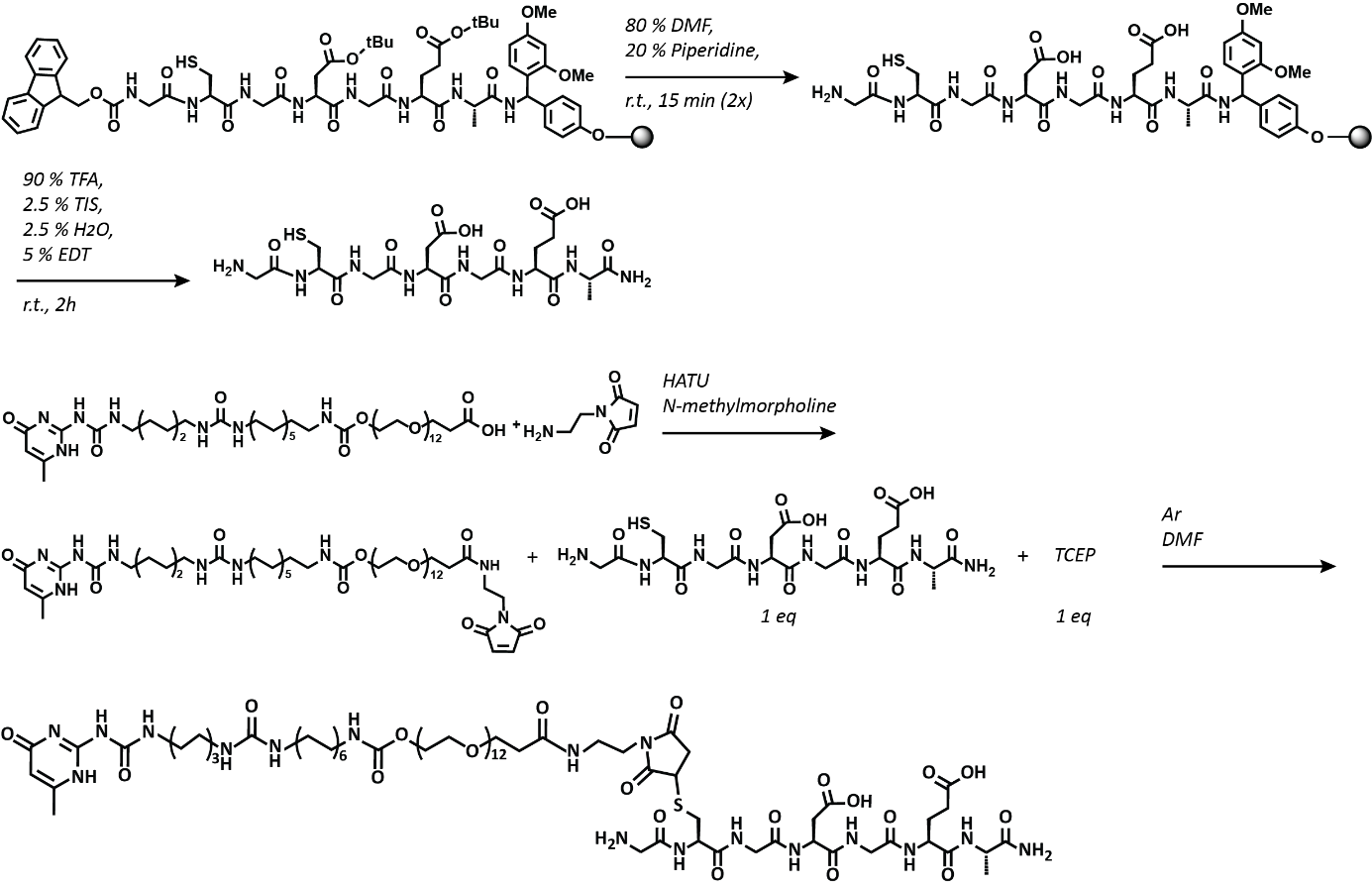


**Scheme S2.** Conjugation of Fmoc SPPS GCGDGEA to a UPy-moiety via maleimide-thiol chemistry, resulting in the supramolecular bioactive additive UPy-GCGDGEA (referred to as UPy-DGEA).


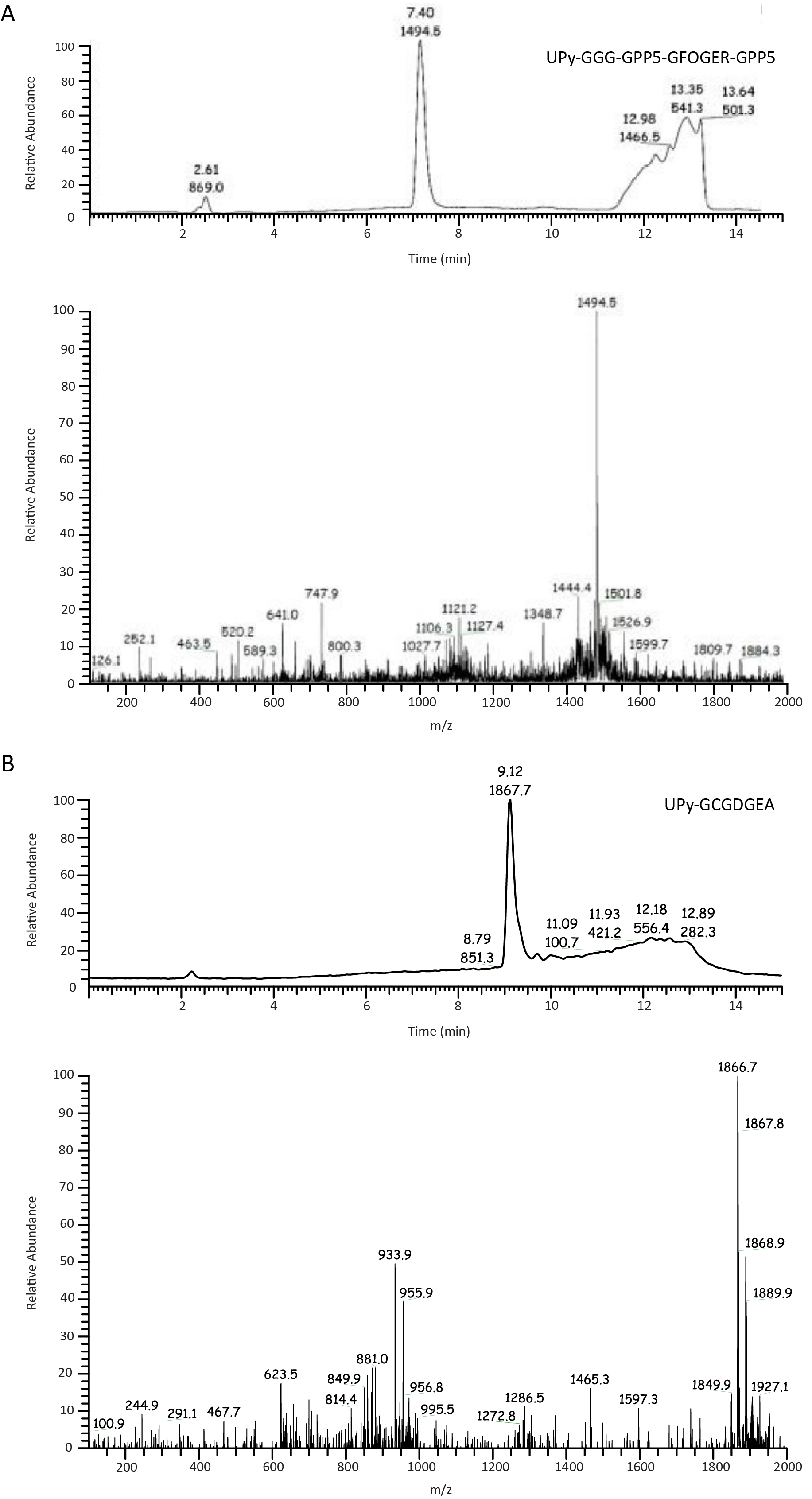


**Figure S1.** LC-MS analysis of UPy-peptide additives. **A)** UPy-GGG-GPP5-GFOGER-GPP5 calculated MW = 4473.35 g mol^-1^, LC-MS(ESI) m/z found 1494.5 [M+3H]^3+^. B) UPy-GCGDGEA calculated MW = 1867.14 g mol^-1^, LC-MS(ESI) m/z found 1867.7 [M+H]^+^, 933.9 [M+2H]^2+^.


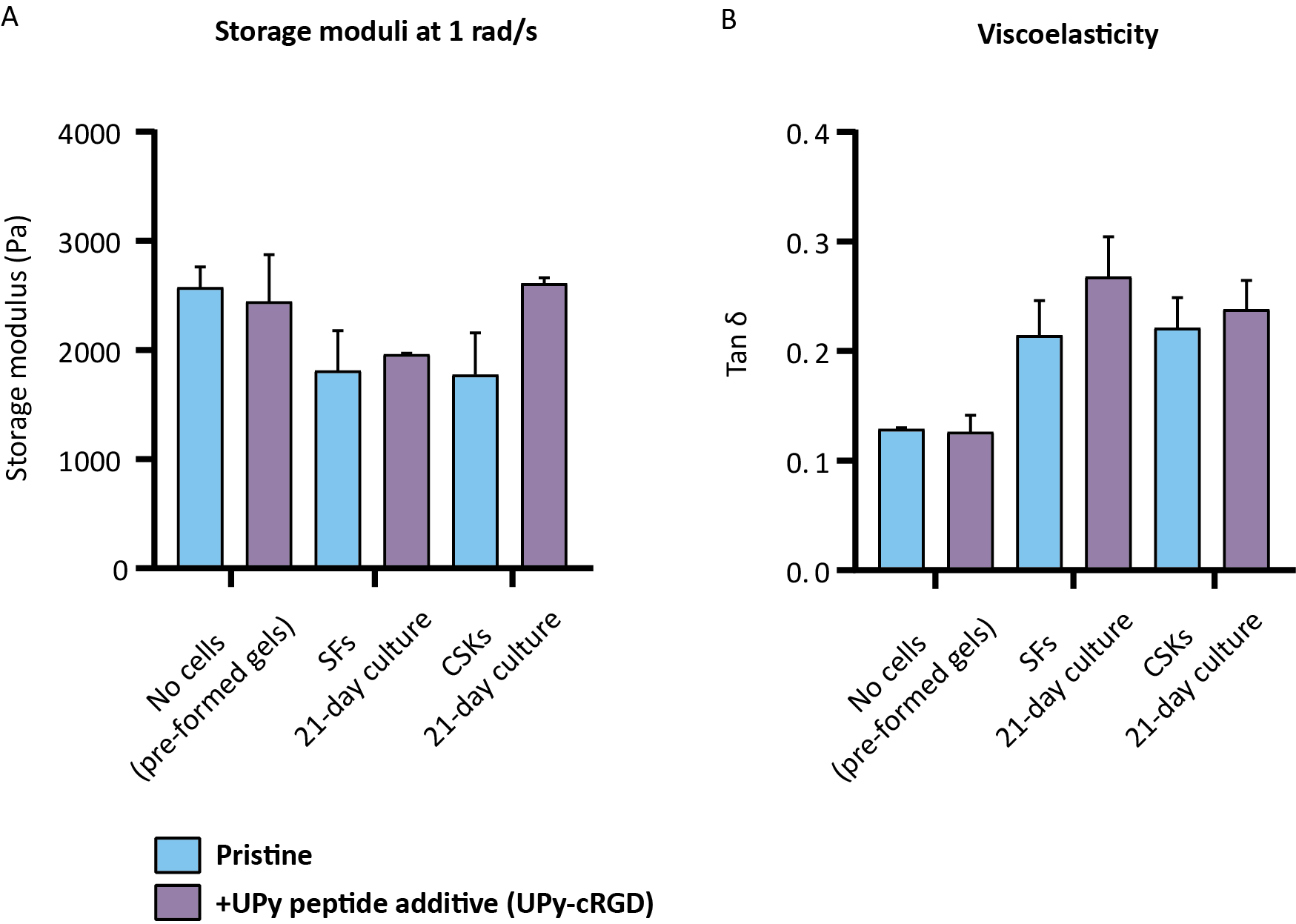


**Figure S2.** Mechanical properties of a pristine hydrogel versus a hydrogel with an incorporated UPy peptide additive (UPy-cRGD). Condition ‘no cells’ are pre-formed hydrogels, prepared in an identical manner as the SFs and CFKs conditions, stored in medium for 24 h at 37 °C before measurement. Condition ‘SFs’ and ‘CSKs’ are hydrogels with encapsulated cells treated towards SFs or CSKs during a 21-day 3D culture. **A)** Storage moduli measured at 1 rad s^-1^ and 1% strain, sample size n=2, mean ± standard deviation. **B)** Tan(delta) values of the hydrogels measured at 1 rad s^-1^ and 1% strain, sample size n=2, mean ± standard deviation. [51]

**Rheological measurements**
A discovery hybrid rheometer (DHR-3, TA Instruments) was used for all the rheological measurements of supramolecular solutions (formations measurements in SI) and pre-formed hydrogels.

*Measuring pre-formed hydrogels without cells*

Hydrogels were made via above mentioned protocol inside a polystyrene 96 well F-bottom cell culture microplate (Greiner bio-one, 655180). After formation, the gels were left 24 hours incubated in PBS at 37 °C. For measurement, gels were transferred onto the peltier plate and a flat stainless-steel plate-plate geometry (diameter = 8 mm) was used. The geometry was slowly lowered, to prevent sample damage, until the sample completely filled the geometry, resulting in a gap height of 625 – 1050 µm. Low viscosity oil (47 V 100, RHODORSIL®) was applied to seal the gap around the hydrogel to minimize evaporation or drying during the measurements performed on 37 °C.[51]

*Measuring pre-formed hydrogels with cells*

Hydrogels were made via the below mentioned cell encapsulation protocol, inside a polystyrene 96 well F-bottom cell culture microplate (Greiner bio-one, 655180). Prior to the measurement, during cell culture, the gels were stored embedded in medium inside a cell culture incubator at 37 °C, 21% O_2_ and 5% CO_2_ for 17 or 21 days, respectively. On the day of the measurement the medium on top of the gels was removed and the gels were washed 1x with PBS. Subsequently the gels were transferred to the rheometer and a flat stainless-steel plate-plate geometry (diameter = 8 mm) was used. The geometry was slowly lowered, to prevent sample damage, until the sample completely filled the geometry, resulting in a gap height of 625 – 1050 µm. Low viscosity oil (47 V 100, RHODORSIL®) was  applied to seal the gap around the hydrogel to minimize evaporation or drying during the measurements performed on 37 °C.[51]


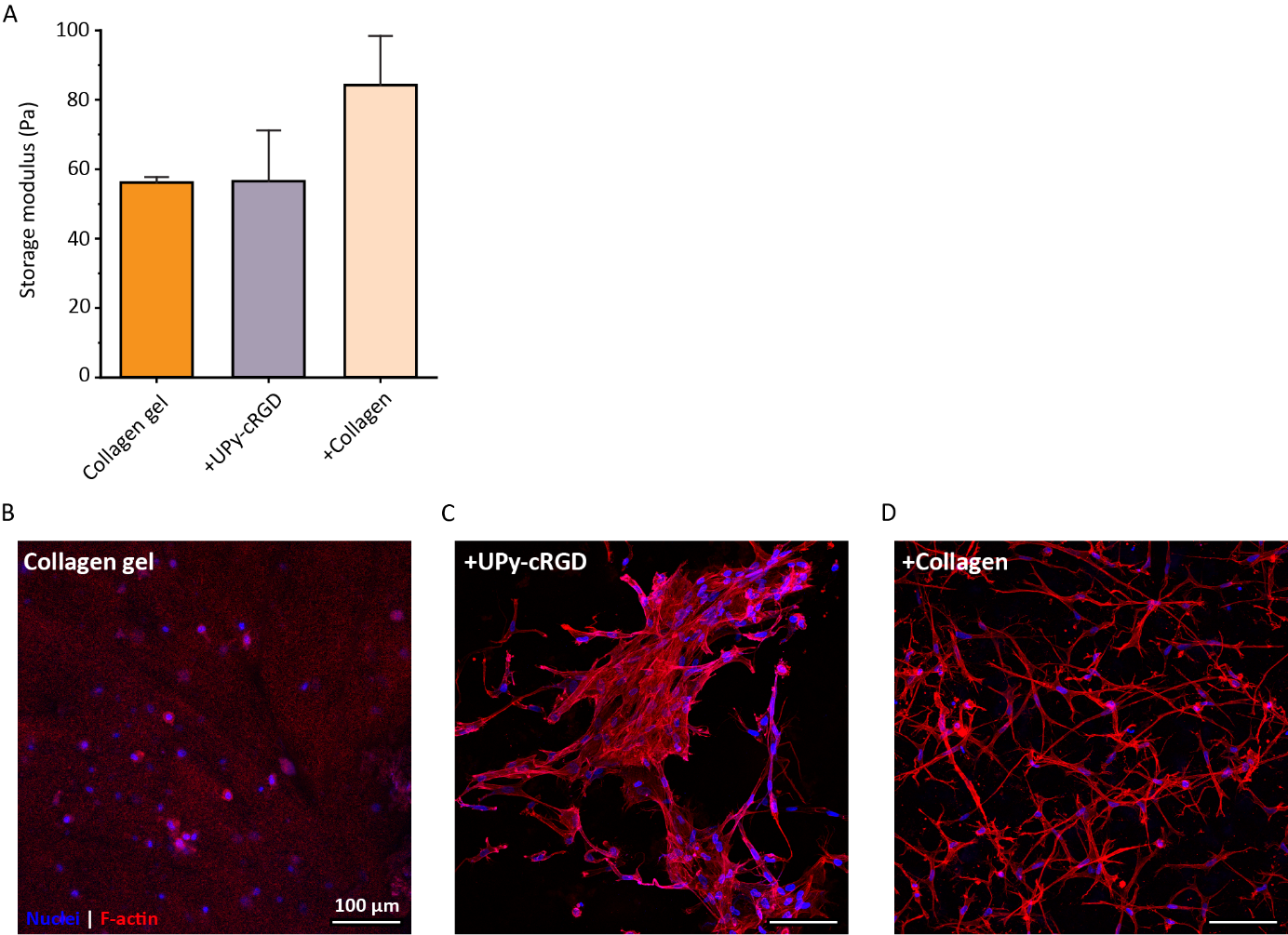


**Figure S3.** Additional comparative study with a full natural collagen hydrogel to evaluate the performance of synthetic hydrogels. **A)** Storage moduli measured at 1 rad s^-1^ and 1% strain, sample size n=2, mean ± standard deviation. For the full natural collagen hydrogel it is not possible to reach a higher storage modulus, therefore the polymer concentrations within the synthetic and hybrid hydrogel are adjusted to obtain hydrogels with storage moduli in a similar range. **B)** Immunohistochemical staining of the nuclei (blue) and F-actin (red) showing round shaped keratocytes when encapsulated within the full natural collagen hydrogel and cultured for 3 days. **C)** Elongated keratocytes encapsulated within the synthetic hydrogel functionalized with an UPy peptide additive (+UPy-cRGD). **D)** Elongated keratocytes encapsulated within the hybrid hydrogel functionalized with natural collagen (+Collagen). [68]

Hydrogels were prepared with the similar procedure as described above (1.25 wt/v% hydrogels). Except for the gelation time, the 0.3 wt/v% hydrogels were incubated for 2 hours (instead of 1 hour) at 37 °C to allow proper gelation. Upon encapsulation of cells within the 0.3 wt/v% hydrogels, the well plates were turned up-side-down during the gelation time. The full collagen hydrogel was prepared according to the provided procedure of the manufacturer (Gibco, Collagen I bovine, A1064401). [68]

**Figure S4.** Quantification of the concentration of keratocytes (SFs) within the hydrogels. **A)** Sample size n=3, three images used per hydrogel, counted cells were corrected for the volume of the z-stack. Initially started with 100 cells/µL for the gels loaded with PKs treated towards SFs. **B)** A selection the of images used for the quantification of the SFs concentration within the various hydrogels. Images show an immunohistochemical staining of the nuclei (magenta) and F-actin (green) of the SFs cultured within the various hydrogels.


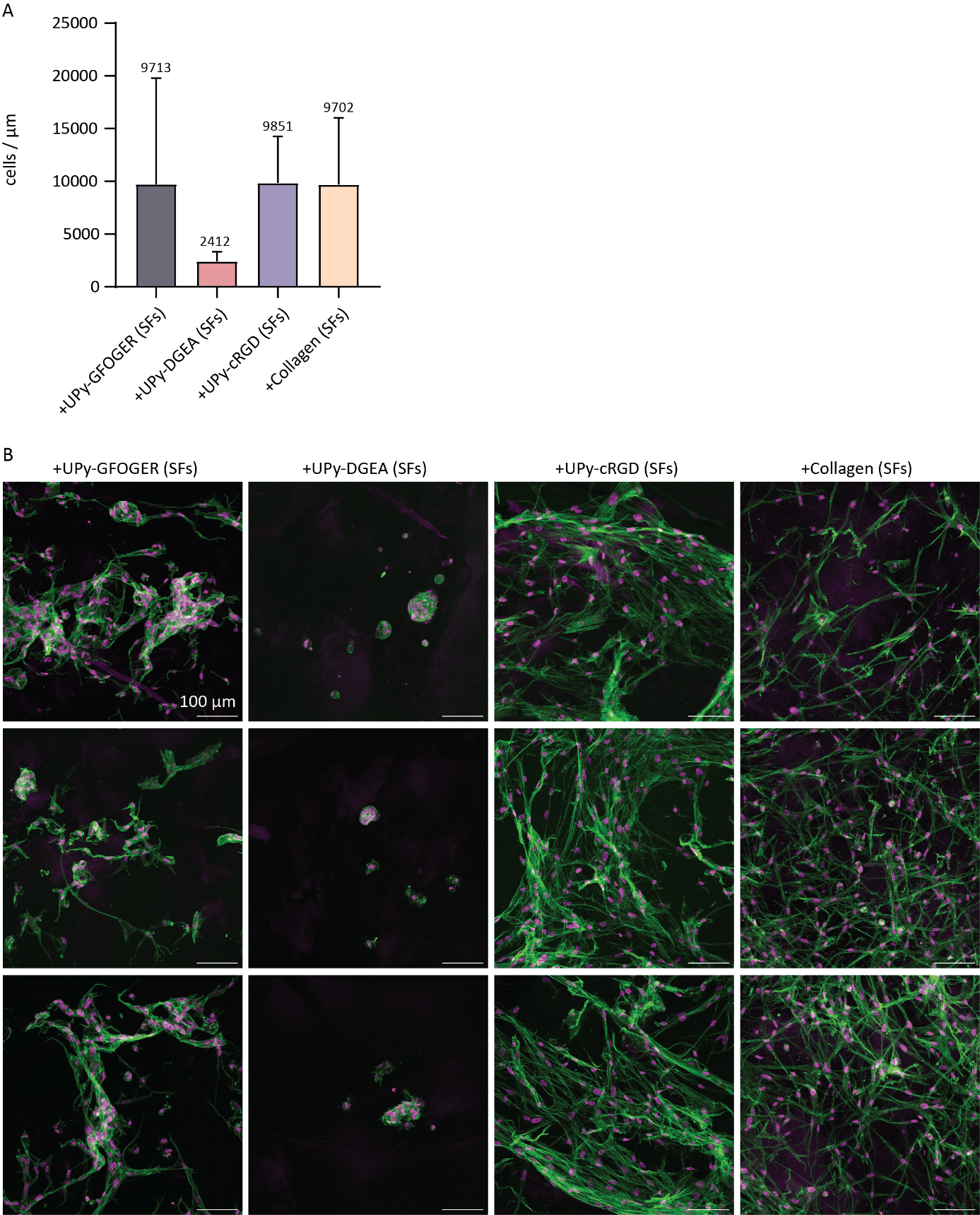

Supplement: Multimedia component 1 [file mmc1.docx]
